# Implementation of Society for Hospital Epidemiology of America (SHEA) and Centers for Disease Control and Prevention (CDC) Outbreak Response Training Program tools to develop a dedicated coronavirus disease 2019 (COVID-19) hospital in Lucas County, Ohio

**DOI:** 10.1017/ice.2021.158

**Published:** 2021-04-15

**Authors:** Jennifer A. Hanrahan, Joel A. Kammeyer, Deana Sievert, Brenda Naylor, Sadik Khuder, Brian Kaminski

**Affiliations:** 1 Division of Infectious Diseases, University of Toledo, Toledo, Ohio; 2 ProMedica Health Systems, Ohio; 3 Metro ProMedica Health Systems, Toledo, Ohio; 4 Department of Medicine, University of Toledo, Toledo, Ohio; 5 ProMedica Health Systems, Toledo, Ohio

## Methods

A team was developed in January 2020 that included systemwide key leaders using the ORTP guidance document as a framework to identify internal stakeholders.^1^ Tool kits available on the ORTP Tool Kits website were used to guide planning.^4^

BayPark Hospital (BPH), a 77-bed acute-care hospital, was designated as the COVID-19 hospital for the ProMedica system, a 13-hospital system in lower Michigan and northwestern Ohio that included 1,472 inpatient beds. This approach preserved other ProMedica hospitals for care of non–COVID-19 patients. Patients requiring hospitalization presenting to other ProMedica hospitals with suspected or confirmed COVID-19 were transferred to BPH, and those presenting to BPH not suspected of having COVID-19 were transferred out.

Overall, 10 staff received intense training on donning and doffing procedures and personal protective equipment (PPE) conservation strategies. These trainers were trained in person and demonstrated correct technique prior to training others. Observers were used for all donning and doffing and patient care to monitor procedures and identify breaches. New staff working at BPH received just-in-time training and demonstrated proper donning and doffing prior to patient care.

A dedicated team of physicians were recruited to develop experience and expertise in the treatment of COVID-19 patients. Algorithms were developed for emergency departments (ED) to maximize diagnostic capabilities while rationing severe acute respiratory coronavirus virus 2 (SARS-CoV-2) testing. Patients evaluated in EDs throughout the ProMedica system suspected of having COVID-19 underwent testing and were reviewed by a dedicated team prior to transfer to BPH.

BPH employees received N-95 respirator fit testing and training on PPE donning and doffing. Safety observers were assigned to COVID-19 units. Protocols were established to limit the type and number of healthcare workers (HCWs) entering rooms of COVID–19 patients, limiting utilization of PPE. Most physician visits were converted to telemedicine encounters.

## Results

The first patient with COVID-19 was admitted to BPH on March 14, 2020. From March 14 to May 30, 2020, 345 patients were admitted to BPH with suspected SARS-CoV-2 infection. Of these, 281 were confirmed positive for SARS-CoV-2 by polymerase chain reaction (PCR) testing, and 3 were designated as probable cases in accordance with the CDC definition. Among them, 80 were critically ill and required care in an intensive care unit (ICU). No symptomatic cases of SARS-CoV-2 infection occurred among HCWs at BPH, and no employees reported exposures to patients due to lack of or improper PPE use.

Among HCWs at other ProMedica hospitals, 3 cases of COVID-19 were reported to the occupational health departments. None of these were due to occupational exposure. Absenteeism related to COVID–19 was minimal.

## Discussion

The ProMedica experience at BPH illustrates that a smaller hospital within a larger health system dedicated entirely to the care of COVID-19 patients provided safety for HCWs at a time that many hospitals reported high rates of transmission to HCWs.^5,6^ We implemented strategies to conserve PPE early on and developed expertise in recognition of COVID-19, including less common symptoms later reported by the CDC.^7^ Successful implementation of this strategy depended on prepandemic planning, and the initial meetings to discuss development of a hospital dedicated to COVID-19 patients were held 2 months prior to the first case being identified in Lucas County, Ohio. Planning started at a time when it was not yet clear that COVID-19 was going to be a pandemic and required buy-in from leaders in the hospital. Although many health systems established COVID-19–only hospitals as the pandemic progressed, our strategy was informed by the ORTP planning tools, which helped to shape the early planning process.

When the first patient was hospitalized at BPH, HCWs were ready to care for that patient and were confident in their training. Having a cadre of nurses, respiratory therapists, and physicians trained in advance to care for patients with COVID-19 was critical in alleviating anxiety, which was expressed by many HCWs throughout the United States. Our model allowed safe care for patients and optimized HCW safety.

The advantages of this model were that PPE use was consolidated, and staff became adept at donning and doffing PPE correctly and identifying best practices to conserve PPE. Concentrating training among a core group of HCWs who then trained others allowed us to quickly train all needed personnel. Having most patients with COVID-19 in one facility allowed patient care protocols to be standardized, bolstered diagnostic and testing capabilities, and facilitated communication among staff.

The main disadvantages of this model of care were that we did not anticipate that patients in Michigan would not be allowed to be transferred to Ohio. This meant that we needed to rapidly duplicate our process in Michigan. Also, transferring critically ill patients at a time when rapid testing was not available meant that patients were transferred with suspected rather than confirmed illness.

Our experience suggests that a hospital dedicated entirely to a novel infection may be a useful strategy for healthcare systems in pandemic planning. This strategy should be considered by large health systems in their advanced planning for future pandemics.
